# Rendu-Osler disease, a cause of recurrent epistaxis: a case report

**DOI:** 10.25122/jml-2025-0070

**Published:** 2025-04

**Authors:** Raluca Oana Pulpă, Cătălina Voiosu, Ruxandra Oana Aliuș, Irina Gabriela Ioniță, Andreea Rusescu, Răzvan Hainăroșie, Viorel Zainea

**Affiliations:** 1 ENT Department, Carol Davila University of Medicine and Pharmacy, Bucharest, Romania;; 2 ENT Department, Prof. Dr. D. Hociota Institute of Phonoaudiology and Functional ENT Surgery, Bucharest, Romania

**Keywords:** Rendu-Osler-Weber, epistaxis, telangiectasia

## Abstract

Rendu-Osler disease is a rare, autosomal dominant vascular malformation disorder with diverse clinical manifestations. It commonly presents with recurrent epistaxis, iron deficiency, and secondary anemia. The condition affects small vessels in the nasal, oral, and gastrointestinal mucosa, the skin of the face, lips, and fingertips, and solid organs. This article discusses general and specific manifestations of the disease, along with its general and particular management. A case presentation is included to demonstrate that treatment must be individualized, taking into account all aspects of the condition, including local and systemic manifestations, comorbidities, and the patient’s chronic treatment.

## INTRODUCTION

Hereditary hemorrhagic telangiectasia (HHT), also known as Rendu-Osler-Weber syndrome, is a rare vascular malformation disorder inherited in an autosomal dominant pattern and characterized by various clinical manifestations. It is a rare disorder that affects the formation of small blood vessels, causing telangiectasias and arteriovenous malformations in 1 of 10,000 individuals [1]. Other studies report a prevalence as high as 1 in 5,000, likely due to underdiagnosis [2]. The highest prevalence rates, approximately 1 in 1,330, have been observed in Afro-Caribbean populations residing in Bonaire and Curaçao [2-4]. According to statistical data, hereditary hemorrhagic telangiectasia is the second most frequent hereditary bleeding disorder in the world, after von Willebrand disease.

The disease was independently described for the first time in the 19^th^ century by Henri Jules Louis Marie Rendu, William Osler, and Frederick Parkes Weber. In 1896, Rendu published a paper describing a syndrome characterized by recurrent epistaxis and telangiectasias, different from hemophilia [5]. Later, in 1901, Osler reported other similar cases [6], as did Weber in 1907 [7]. It is a genetic, multisystem disorder characterized by abnormal angiogenesis [8]. Mutations in three genes have been responsible for creating an unstable state between pro-angiogenetic and anti-angiogenetic factors [9]. Consequently, these patients develop significant arterial and venous malformations and telangiectasias with cutaneous and mucosal disposition [10-13].

In Rendu-Osler-Weber syndrome, capillaries of the skin, mucous membranes, and specific organs are not present, and direct communication between dilated arteries and veins is present. These vascular malformations are highly fragile, and the slightest trauma can cause bleeding [14]. The most frequent clinical manifestation of a patient diagnosed with hereditary hemorrhagic telangiectasia is recurrent epistaxis, sometimes severe, with systemic repercussions like chronic anemia (almost always encountered with these patients) and iron depletion [15]. Most patients with Rendu-Osler-Weber syndrome need iron supplementation and repeated blood transfusions throughout their lives.

Recurrent epistaxis is the most common clinical manifestation, occurring in up to 95% of patients due to the presence of nasal mucosal telangiectasias [16]. The clinical overall picture includes blood vessel dilations at the skin site and oral and gastrointestinal mucosa. Solid organs, like the lungs, liver, and brain, are affected by arteriovenous malformation. As it is a genetically inherited disease, family history is also present.

The arteriovenous malformations are visible as red or pink lesions, with a diameter of 0,5–1,00 mm, present all over the body but usually on the lips, tongue, face, fingertips, and nasal, oral, and gastrointestinal mucosa. Larger blood lakes formed by arteriovenous shunts can range from a few millimeters to centimeters, are prone to rupture, and can cause moderate or severe bleeding. Telangiectasias differ from petechiae as they fade when pressure is applied and refill and reappear immediately after the pressure stops [4,13].

Because of the diverse manifestations and multiple affected sites, patients affected by Rendu-Osler-Weber syndrome may present to different physicians, such as emergency medicine specialists, family doctors, and surgical or medical specialists. Many cases receive a delayed or misinterpreted diagnosis as the condition is not common, and heterogeneous manifestations characterize it [17,18].

To facilitate the diagnosis, the medical community established in 2000 the Curaçao criteria. It contains four elements:

- Epistaxis: recurrent, mild, or severe;
- Telangiectasias: multiple lesions at specific sites (oral cavity, lips, fingers, nasal mucosa);
- Visceral arteriovenous malformations: gastrointestinal, liver, lungs, brain;
- Family history: a first-degree relative with the disease;

The diagnosis is definite if three or more criteria are present, possible or suspected if only two criteria are present, and unlikely if only one element is encountered. Recurrent episodes of epistaxis cause secondary anemia and low levels of iron in the blood. However, an endoscopic gastrointestinal evaluation is mandatory when the hemoglobin levels are discordant with the severity and frequency of nosebleed episodes. The most frequent gastrointestinal bleeding sites are found in the stomach and duodenum, so an esophagogastroduodenoscopy is usually sufficient to identify telangiectasia. Large bleeding telangiectasias may be cauterized during endoscopy to control hemorrhage [19-21]. Merely 10% of patients with hereditary hemorrhagic telangiectasia and hepatic vascular malformations are symptomatic without catastrophic consequences. The liver vascular malformities are identified using noninvasive investigations like CT, MRI, or Doppler ultrasound. For investigating pulmonary arteriovenous malformations, transthoracic contrast echography is recommended. The management of these lesions is interventional, transcatheter embolization, but used only for life-threatening hemorrhages. Angiography is ideal for diagnosing cerebral arteriovenous malformations. Other techniques that can be used for diagnosis have a risk of stroke or even death [1].

## CASE REPORT

A 70-year-old male patient, diagnosed at age 32 with Rendu-Osler-Weber syndrome, presented to our emergency department with recurrent nasal bleeding. Typically, he managed these episodes at home using nasal compression, topical vasoconstrictors, or hemostatic sponges (Gelaspon type). However, the current episode was more severe and refractory to these measures.

**Figure 1:**
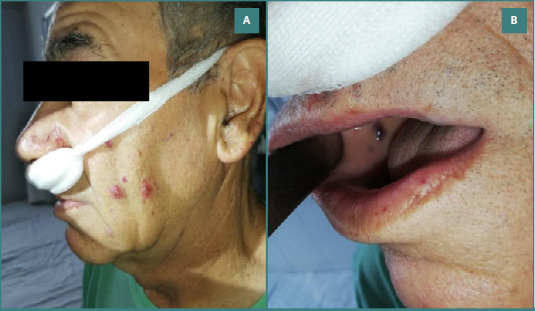
Dermal and oral mucosal telangiectasis. A, Multiple telangiectatic lesions on the facial skin, particularly on the cheek area; B, Telangiectasias visible on the oral mucosa, especially on the lips and buccal surface.

The initial evaluation revealed a pale patient with telangiectasis of the dermal and mucosal surfaces, including the lips, tongue, and jugal mucosa (Figure 1 AB).

**Figure 2:**
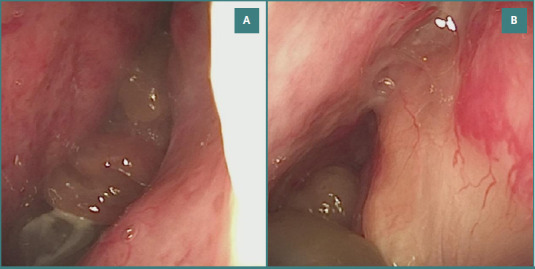
Polypoid formations and nasal mucosa telangiectasis. A, Polypoid masses partially obstructing the nasal cavity, surrounded by telangiectatic vascular lesions; B, Detailed view of nasal mucosa highlighting fragile telangiectatic vessels adjacent to polypoid structures.

He was actively experiencing bilateral epistaxis at the time of examination. Vital signs revealed a blood pressure of 165/90 mmHg and a heart rate of 90 bpm. The endoscopic examination revealed multiple bleeding sites on both nostrils and disseminated vascular lesions all over the pituitary region. Furthermore, the nasal airway passage was blocked by polypoid formations (Figure 2 AB).

Previous digestive endoscopies, esophagogastroduodenoscopy, and colonoscopy revealed vascular lesions within the gastrointestinal mucosa. Although no first-degree relatives had been diagnosed with Rendu-Osler-Weber syndrome, the patient fulfilled three of the four Curaçao criteria, confirming the diagnosis. The patient's medical history included hypertension (managed with antihypertensives), a mechanical aortic valve prosthesis for aortic insufficiency, and long-term anticoagulation with acenocoumarol for thrombosis prevention. Laboratory evaluation revealed anemia and iron deficiency, as summarized in Table 1.

**Table 1:** Laboratory results/reference values

Laboratory parameters	Case patient	Reference value
Leukocytes	4.5 10^3^/ul	4.00 – 9.00 10^3^/ul
Red blood cells	3.49 10^6^/ul **↓**	4.5 – 6.10 10^6^/ul
Hemoglobin	9.6 g/dl **↓**	13.00 – 18.00 g/dl
Hematocrit	29.2 % **↓**	39.00 – 52.00 %
VCM	83.7 fl	80.00 – 95.00 fl
HCM	27.5 pg	26.00 – 33.5 pg
CHCM	32.9 g/dl	31.00 – 35.5 g/dl
Erythrocyte distribution width	21.9 % **↑**	10.00 – 16.00 %
Platelets	0.158 %	0.15 – 0.50 %
Serum iron level	18,6 mg/dl **↓**	>40 mg/dl
PT	27.0 s **↑**	10.00 – 15.00 s
INR	2.41 **↑**	0.80 – 1.26

MCV, mean corpuscular volume; MCH, mean corpuscular hemoglobin; MCHC, mean corpuscular hemoglobin concentration; PT, prothrombin time; INR, international normalized ratio; **↓**, decreased value; **↑**, increased value.

The results confirmed secondary anemia and iron deficiency, consistent with the previous blood test history. The patient was under regular hematology follow-up, receiving periodic iron supplementation and transfusions as needed. He was admitted, and bilateral nasal packing with Merocele was performed after removing all the clots in the nose. The oral treatment with anticoagulants was interrupted, and heparin treatment with low molecular weight was initiated. After 3 days, hemoglobin levels and coagulation parameters normalized.

The nasal packing was maintained for 72 hours and removed under endoscopic control while the patient was sedated. Minor bleeding was controlled with bipolar cautery, and resorbable hemostatic strips were applied. No recurrent bleeding occurred, and oral anticoagulation was resumed.

During one year of follow-up, nasal bleeding episodes became less frequent despite the progression of nasal polyps, which eventually occupied both nasal passages. This reduction in bleeding was possibly due to a tamponade effect exerted by the polypoid masses.

Given the patient’s cardiovascular history, anticoagulant therapy, and underlying disease, surgical removal of the nasal polyps was carefully planned with informed consent. Acenocumarol treatment was stopped for 5 days before the intervention, as the cardiology specialist indicated, and was replaced with low molecular heparin administered subcutaneously. We performed the intervention under general anesthesia, removing the polypoid masses using the microdebrider. Intraoperative bleeding was comparable to that observed in patients without hereditary hemorrhagic telangiectasia and was managed with bipolar cautery and hemostatic powder. Merocele nasal packing was applied and removed after 72 hours. The evolution was satisfying; the low-volume bleeding in the first days after removing the nasal packing was controlled using intranasal tranexamic acid spray. Ninety-four hours after the surgical intervention, acenocoumarol treatment was reinitiated, while low molecular weight heparin was continued until the international normalized ratio (INR) reached 2.

## DISCUSSION

We chose to present this particular case because it is representative of hereditary hemorrhagic telangiectasia, fulfilling three of the four diagnostic criteria. However, the patient's comorbidities and long-term treatment with anticoagulants raised difficulties in the general management of the disease.

Rendu-Osler-Weber syndrome is a condition that needs multidisciplinary follow-up and treatment. The medical team includes ENT specialists, hematologists, gastroenterologists, lung specialists, and neurologists. Usually, the first specialist to interact with the patient is an otorhinolaryngology specialist, as the most prominent manifestation is nasal bleeding. All of the patients manifest at least one episode of nasal bleeding before the age of 40, and more than half of them have recurrent epistaxis, becoming more frequent and severe as the person ages [2].

Hereditary hemorrhagic telangiectasia is a condition with a high risk of venous thromboembolism due to blood stagnation at the level of peripheral vascular lakes. Still, patients permanently suffer from hemorrhagic events. In cases with no other complications except the classical presentation of the syndrome, the general recommendation is to avoid using anticoagulant agents and antiplatelet therapy [22,23]. In the case of our patient, the cardiac valvular pathology imposed the long-term use of antiplatelet medication as the thrombotic risk was much higher in comparison with other patients with Rendu-Osler-Weber syndrome and the general population.

In the future, a new surgical intervention for chronic polyposis rhinosinusitis may be needed. In this case, the hemorrhagic risk is higher due to the underlying diseases and chronic anticoagulant treatment. Biological treatments approved for patients with asthma and nasal polyposis may help reduce the number of surgical interventions and increase the surgery-free period.

There are three lines of treatment: conservative therapies, medical therapies, and surgical procedures. Conservative therapies, such as nasal humidification with saline solutions and topical emollients, seem to reduce episodes of epistaxis [23]. Systemic estrogen and tamoxifen, an anti-estrogen agent, have yielded promising results. Bevacizumab, a vascular endothelial growth factor (VEGF) inhibitor, is applied topically or injected submucosally and tends to reduce the severity of epistaxis [24]. Beta-blockers, like timolol or propranolol, proved to have an anti-angiogenetic effect when administered topically and reduced the severity and frequency of epistaxis, decreasing the need for blood transfusion. Tranexamic acid works as an anti-fibrinolytic agent and has been shown to shorten epistaxis episodes. N-acetylcysteine, an oxygen-free radical scavenger, is also under investigation due to the potential role of oxidative stress in the development of vascular malformations. In terms of local nasal treatment, in this particular case, we noticed a satisfying result with the use of tranexamic acid in a spray form. This treatment reduces the frequency and severity of episodes of epistaxis.

As surgical procedures, the most frequently used are bipolar coagulation, laser photocoagulation, and coblation using argon plasma. Argon plasma offers the advantage of operating at lower temperatures (40–70°C), reducing the risk of deep tissue injury. Septodermoplasty is a procedure that removes the affected septal mucosa and replaces it with buccal mucosal grafts [25]. The disadvantage is that telangiectasias also tend to develop on the skin graft. It is important to note that embolization of the sphenopalatine, facial, or maxillary arteries, external radiotherapy, and Young's procedure are rarely performed and are reserved for carefully selected cases.

## CONCLUSION

Hereditary hemorrhagic telangiectasia should not be considered an absolute contraindication for anticoagulant and antiplatelet therapies. Still, these agents should be used at the lowest possible doses, considering the risk/benefit ratio. The follow-up and treatment of this disease require a multidisciplinary team. Various therapies have been employed to reduce the severity and frequency of epistaxis episodes, though achieving complete control without compromising quality of life remains challenging. Treatment must be individualized for each patient, considering local and systemic manifestations, comorbidities, chronic treatments, and the need for surgical procedures related to other conditions. Nasal bleeding should be addressed progressively, beginning with conservative measures, followed by medical therapies, and reserving surgery for cases in which these approaches fail.
